# Ocular Chemical Injuries in Western Saudi Arabia: A Study of the Public's Level of Knowledge and Experience

**DOI:** 10.7759/cureus.40724

**Published:** 2023-06-21

**Authors:** Ashjan Bamahfouz, Salah M Bakry, Alhanouf M Alsharif, Salwa Alomeri, Elaf F Alsharif, Osama S Zamzami, Soha Emorsy

**Affiliations:** 1 Ophthalmology, Umm Al-Qura University, Makkah, SAU; 2 Ophthalmology, King Abdullah Medical City, Makkah, SAU; 3 Ophthalmology, Security Forces Hospital, Makkah, SAU; 4 Medicine, Umm Al-Qura University, Makkah, SAU; 5 Ophthalmology, Jeddah Eye Hospital, Makkah, SAU; 6 Intensive Care Unit, King Abdulaziz Medical City Jeddah, Jeddah, SAU; 7 Medicine, Cairo University, Cairo, EGY

**Keywords:** ocular injuries, western region of saudi arabia, saudi arabia, prevention, awareness, knowledge, ocular chemical injuries

## Abstract

Background: Chemical burns are potentially blinding eye injuries and are serious ocular emergencies that necessitate prompt evaluation and treatment.

Aim: This study aimed to evaluate the knowledge and experience of the current practice of ocular chemical injuries among the general population in western Saudi Arabia.

Methods: An electronic self-administrated structured survey was distributed among citizens using social media channels in November 2022.

Results: This survey includes 929 participants from western Saudi Arabia. Most of the participants were more than 20 years old (82.7%), while females represented 82.7%. Most participants reported an inadequate level of awareness about previous hearing of ocular chemical injury terms compared with their level of knowledge, in which the majority (56.62%) showed a good level of understanding. Female participants, participants 20 years old or older, and Saudis corresponded significantly with a good level of knowledge (p-values <0.001, <0.00, and 0.025, respectively).

Conclusion: This study showed a fair level of knowledge compared to awareness, which can be improved by further national studies in the Saudi region. We recommend expanding the studies’ findings and developing suitable interventions, like health awareness campaigns about ocular chemical damage and prompt corrective measures.

## Introduction

Ocular injuries have a significant impact on communities [1,2]. Alkaline and acidic ocular chemical damage are both possible. However, alkaline burns are more common due to the extensive use of alkaline compounds in industrial and household cleaning products; these burns often result in more serious injuries [1,3].

Chemical eye injuries are true emergencies that require immediate recognition and treatment. The primary treatment required limiting tissue damage and maintaining vision through rapid dilution of the chemical agent. The amount of ocular damage is proportional to how far the corrosive substance deviates from pH 7.4, how long it persists in contact with the eye, and how much neutralization is required [4]. Depending on the degree of the injury and when treatment was started, the potential sequelae can range from dry eyes to severe conditions like ectropion, entropion, lagophthalmos, symblepharon, lack of limbal stem cells, corneal opacity, non-healing corneal ulcer, intractable glaucoma, cataract, retinal detachment, and even phthisis bulbi [5-6].

Chemical injuries account for about 7% of workplace-related eye injuries treated in emergency rooms at hospitals in the United States [1,7]. Furthermore, more than 60% of chemical injuries occur in the workplace, 30% arise at home, and 10% are due to an accident outside [1,8]. Up to 20% of chemical eye injuries result in severe vision impairment and facial disfigurement; yet, visual rehabilitation following significant chemical eye damage occurs in only 15% of those who are afflicted [1,7,8].

All community members, especially those who work with chemicals regularly, should be familiar with various types of ocular and periocular injuries [1]. Epidemiological studies are needed to determine the rate at which people are aware of ocular chemical damage and how to respond appropriately [1]. Limited studies have investigated the awareness and practice perception of ocular chemical injuries nationally and internationally [1, 5, 9]. Therefore, these studies need further assessment and investigation in Saudi Arabia.

## Materials and methods

Study design and selection criteria

This descriptive cross-sectional study utilized an electronic, self-structured survey, which was distributed among participants in November 2022. We included all citizens from western Saudi Arabia. However, all participants who refused to complete the survey and citizens from other Saudi regions were excluded.

Sample size

We utilized Epi Info™ 7.1.5 (Center for Disease Control and Prevention; Atlanta, Georgia, USA) for sampling calculation. The minimum possible sample size to achieve an accuracy of ± 5% with a 95% confidence interval (CI) is 384. However, the final sample size of 929 was targeted during data collection.

Questionnaire development and scoring system

In light of the current literature, our survey was adapted from previously published studies [1,5,9]. The questionnaire was classified into two sections. The first part aimed to collect participants’ demographics, including age, gender, nationality, marital status, and educational level. Then, the second part aimed to assess participants’ level of knowledge, awareness, attitude, and experience towards ocular chemical injuries through multiple-choice and closed-ended questions.

The modified Bloom’s criteria were used to estimate knowledge scores [10]. Therefore, the scores were divided into good, moderate, and poor. Accordingly, scores between 80% and 100% were considered good, 50% and 79% were considered moderate, and scores less than 50% were considered poor.

Ethical consideration

This study received ethical approval from the Umm Al-Qura University ethics committee (Institutional Review Board (IRB) code: HAP0-02-K-012-2021-11-841). Furthermore, this survey followed the principles of the Declaration of Helsinki.

Statistical analysis

The data were run through the Statistical Package for the Social Sciences (SPSS), version 23 (IBM, Armonk, NY), spreadsheet after checking for completeness and minor typographical errors. Descriptive statistics were expressed as percentages for categorical variables and mean and standard deviation for continuous variables, and a p-value less than or equal to 5% was considered significant. The categorical variables were computed using the Chi-square test.

## Results

Overall, 929 citizens were surveyed from western Saudi Arabia. Their socio-demographic characteristics are given in Table 1. Participants aged 20 or more were predominant (n = 768) at 82.7% compared with those younger than 20 years. The majority of participants were female (n = 768, 82.7%). Most of the respondents were single. Additionally, concerning their educational degrees, most participants had university degrees. About 47.6% had a positive history of ocular chemical injury. On the other hand, only 4.6% had a positive family history of ocular chemical injury (Table 1).

**Table 1:** Participants' socio-demographic data

Variable	Category	n= 929	(100%)
Age (in years)	Younger than 20	161	17.3
	20 or more	768	82.7
Gender	Male	161	17.3
	Female	768	82.7
Nationality	Saudi	865	93.1
	Non-Saudi	64	6.9
Educational level	Illiterate	18	1.9
	Below university level	164	17.7
	University level	684	73.6
	Other	63	6.8
Marital status	Single	614	66.1
	Married	269	29.0
	Divorced	33	3.6
	Widow	13	1.4
Ever been diagnosed with an ocular chemical injury?	Yes	442	47.6
	No	487	52.4
Family history of ocular chemical injury	Yes	43	4.6
	No	886	95.4

There were five subgroups of ocular chemical injury issues in which the knowledge score was obtained (Table 2). Most participants believed that spillage of a liquid or solid chemical substance in the eye was an example of ocular chemical burns (40.8%). In comparison, only 3.7% believed that oil spillage in the kitchen in the eye was an example of ocular chemical burns. Around 1.3% correctly corresponded to all responses regarding ocular chemical injuries' symptoms. Most participants correctly responded to the first action to take during an ocular chemical injury (59.2%). In addition, the majority corresponded correctly to the action to take in the case of wearing contact lenses during an ocular chemical injury (52.1%).

**Table 2:** Participants' knowledge related to ocular chemical injury

Category	Answers	n= 929	(100%)
Examples of ocular chemical examples	Liquid	379	40.8
	Chemical steam	289	31.1
	Oil	34	3.7
	Home cleaning	227	24.4
Symptoms of ocular chemical injuries	Pain/tears	37	4.0
	Redness	97	10.4
	Can't open eyes	22	2.4
	Blurred vision	46	5.0
	Foreign body sensation	12	1.3
	All of the above	12	1.3
The first step to take in case of the occurrence of ocular chemical injuries	Wash with plenty of water	550	59.2
	Wash with a little water	48	5.2
	Eye coverage	28	3.0
	Going to the emergency department	282	30.4
	Going to the pharmacy to purchase eye drops	21	2.3
The correct action to take in case of wearing contact lenses when an ocular chemical injury occurs	Remove lenses immediately	484	52.1
	Remove lenses if there is eye pain	71	7.6
	Remove lenses in case of suspicion of ocular chemical injuries	172	18.5
	No need to remove lenses	202	21.7
Complications	Cornea and iris damaged	441	47.5
	Corneal perforation	51	5.5
	Eyelid deformities	34	3.7
	Loss of vision	385	41.4
	Myopia/hyperopia	18	1.9

About 47.5% believed that corneal and iris damage is an ocular chemical injury complication, while only about 1.9% believed that myopia and hyperopia are complications of ocular chemical injury.

**Figure 1:**
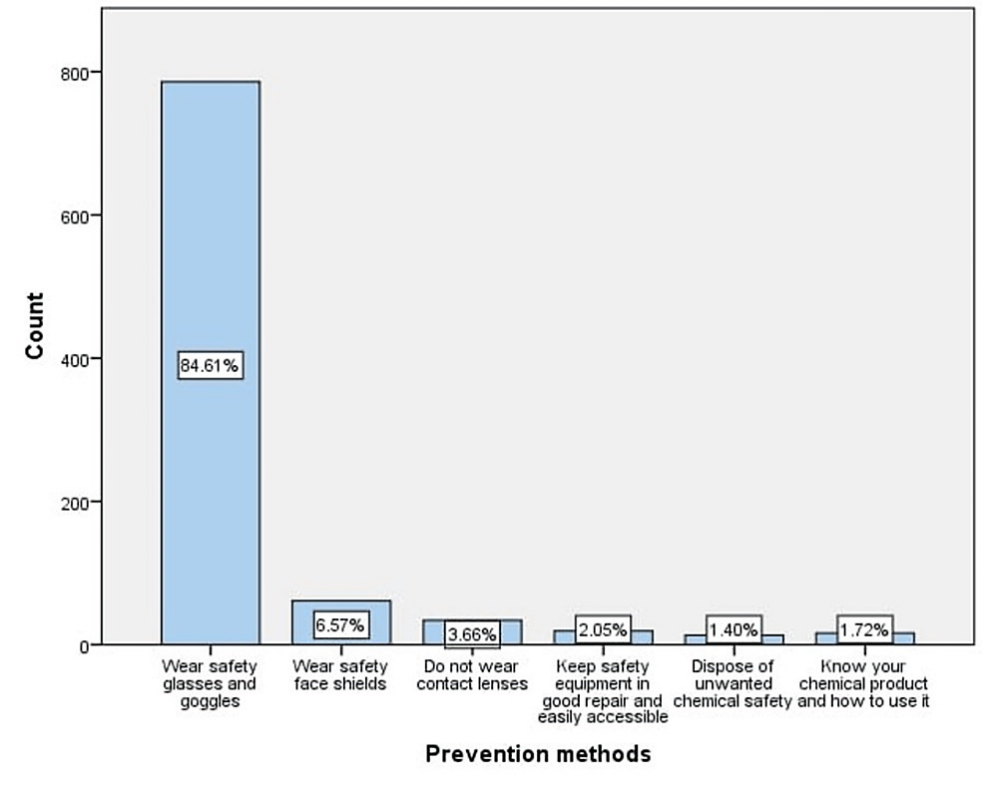
Methods to prevent ocular chemical injuries

**Figure 2:**
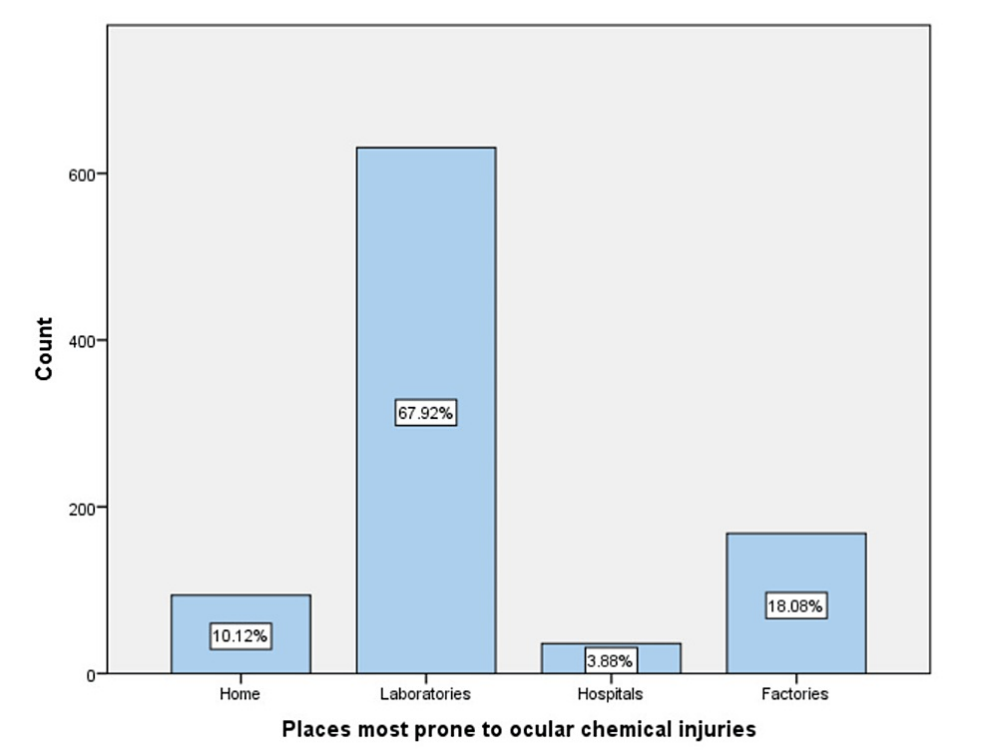
Most prone places for ocular chemical injuries

Most respondents believed that wearing safety glasses and face shields may prevent ocular chemical injuries (84.61 and 6.57, respectively) (Figure 1). On the other hand, laboratories and factories were considered the most prone places for ocular chemical injuries from the participants' point of view (67.92, 18.08, respectively) (Figure 2).

**Figure 3:**
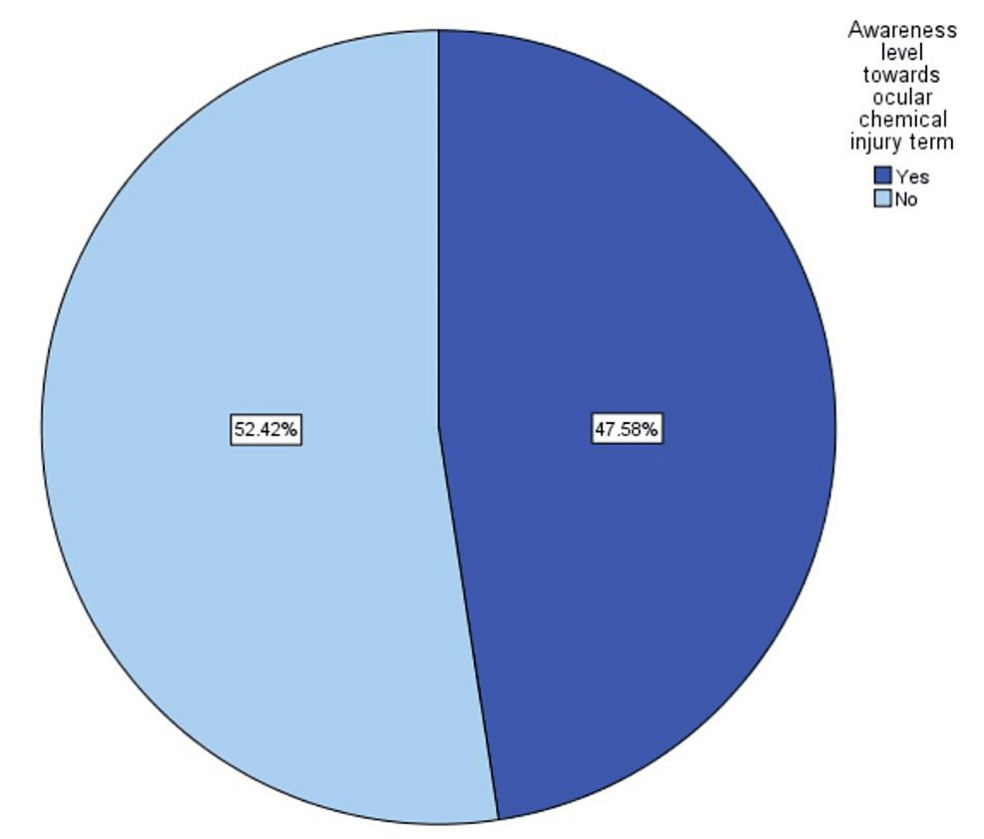
Participants' awareness level of the term "ocular chemical injury"

Concerning the awareness level towards the term "ocular chemical injury" among participants, the majority had a low level of awareness (52.42%) (Figure 3).

**Figure 4:**
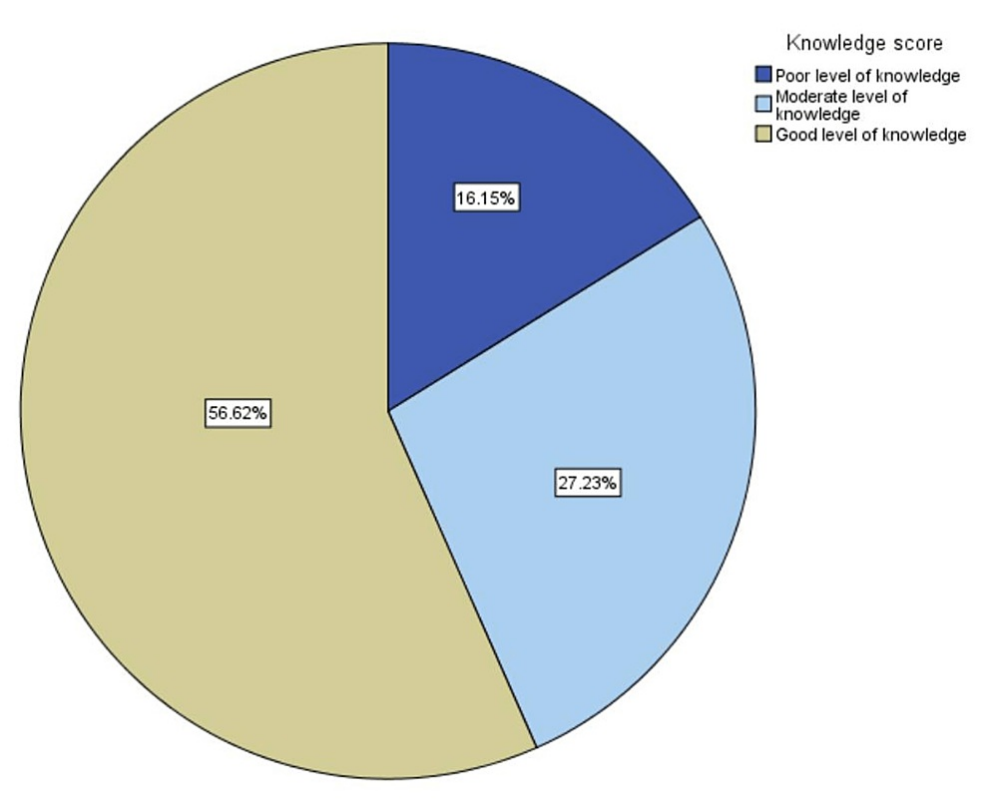
Participants' level of knowledge

However, this is not in line with the level of knowledge. Most participants represent a good to moderate level of knowledge (56.62% and 27.23%, respectively) compared to a poor level of knowledge (16.15%) (Figure 4).

The correlation between participants'' demography and level of knowledge is given in Table 3. Participants who were 20 years of age or older correlated significantly with a good level of knowledge compared with participants less than 20 years old (p-value <0.001). Furthermore, female and Saudi participants corresponded considerably with a good level of knowledge compared with male and non-Saudi participants (p-value <0.001, 0.025, respectively). However, participants' social status shows no significant variation in their level of knowledge (p-value of 0.227).

Participants with university and below-university degrees show significant variation compared with participants with other unlisted educational degrees and illiterate participants (p-value <0.001). A significant association was found between participants with good awareness of ocular chemical injury terms and a good level of knowledge (p-value of 0.001). Moreover, participants with no history or family history of previous ocular chemical injury correspond significantly with those with positive histories (p-value <0.001).

**Table 3:** The correlation between the level of knowledge and participants' demography

Variable	Level of knowledge			p-value
	Good (%)	Moderate (%)	Poor (%)	
Age (in years)				
Less than 20	39.8%	36.0%	24.2%	<0.001*
20 or more	60.2%	25.4%	14.5%	
Gender				
Male	48.4%	24.2%	27.3%	<0.001*
Female	58.3%	27.9%	13.8%	
Nationality				
Saudi	57.3%	27.4%	15.3%	0.025*
Non-Saudi	46.9%	25.0%	28.1%	
Marital status				
Single	57.8%	25.9%	16.3%	0.227
Married	55.8%	30.5%	13.8%	
Divorced	48.5%	24.2%	27.3%	
Widowed	38.5%	30.8%	30.8%	
Educational level				
Illiterate	5.6%	16.7%	77.8%	<0.001*
Below university level	53.7%	28.7%	17.7%	
University level	59.5%	26.8%	13.7%	
Other	47.6%	31.7%	20.6%	
Ever heard about ocular chemical injuries?				
Yes	62.9%	23.5%	13.6%	0.001*
No	50.9%	30.6%	18.5%	
Past history of ocular chemical injuries				
Yes	11.6%	25.6%	62.8%	<0.001*
No	58.8%	27.3%	13.9%	
Family history of ocular chemical injuries				
Yes	33.8%	29.4%	36.8%	<0.001*
No	58.4%	27.1%	14.5%	

## Discussion

Vision is among the most fundamental human functions. Therefore, a chemical injury's impact on vision might significantly affect the quality of life [1]. This study aims to assess public awareness and practice perceptions of ocular chemical injuries. Most of our participants were over the age of 20, and the majority were female, representing a good and moderate level of knowledge.

A recent study spotlighted the knowledge of emergent management of ocular chemical events among participants who are managing the casualty, in which they grouped participants into two main groups: the first group was medical trainees, while the second group was workplace supervisors and family members [5]. Respectively, participants from group one showed a higher level of understanding and practice than those from group 2 [5]. Similarly, another study demonstrated good knowledge and practice among the general population [1]. Conversely, a recent Saudi survey-based study among the Asser's general population reported a poor understanding of ocular chemical injuries [9], which could be explained by the fact that the participants mostly used the internet and other sources to gather their knowledge. If this is not specialized or was not taught by professionals, it may mislead the reader [9]. Furthermore, doctors played a smaller role than anticipated in giving first aid advice, which should be improved [9].

About 47.6% of participants in this study had a previous history of ocular chemical events. Our participants with a prior history are more significant than the two population-based studies conducted in Saudi Arabia, in which 8.1% and 15.7% had a previous history, respectively [1,9].

Our participants were unaware of symptoms of ocular chemical injuries, while most corresponded correctly to eye redness. This is in concordance with a study from India [5]. However, this is not in line with the Saudi research, in which constant eye pain shows the highest response rate as a sign of ocular chemical injuries among participants [9].

Immediate washing and neutralizing the chemicals with water is always recommended as the first corrective step in chemical ocular injuries to minimize tissue damage and safeguard vision [1,11,12].

This study reported that purifying the eye with plenty of water, followed by immediate emergency department evaluation, was the first action to take in cases of ocular chemical events. This strongly agrees with a Saudi study's findings [1].

Strengths and limitations

Our study is considered the first to the authors' knowledge that discusses the general population's awareness and understanding of chemical ocular injury events in western Saudi Arabia, along with a large sample size. However, given that this was an online survey-based study, there are some potential limitations to this research. The selection bias is the main factor preventing these results from being broadly applied. The results are biased because those with higher levels of education (who might fill out the survey) were included in the study since we employed an electronic questionnaire. Furthermore, not all Saudi Arabian regions were represented in the results of our research.

## Conclusions

An ocular chemical injury resulting in vision loss is a significant condition that can adversely impact an individual's quality of life, particularly via job loss and greater dependency on others. The current study determined that public knowledge regarding chemical eye injuries was satisfactory, as the majority had good knowledge.

However, public comprehension regarding first aid for chemical damage can be further improved through periodic health education programs, a greater effort by healthcare workers to explain the main protective measures that can be taken in the event of an injured chemical eye, and incorporating first aid into study courses.
